# Died

**Published:** 1864-02

**Authors:** 


Died—In Washington, D. C., February 5th, Dr. William H. Butler, of this city, who has been connected with the army for the past three years and with Armory Hospital at the time of his death. Dr. Butler was well known to many of our readers, and the present number contains an article from his pen. He has been suffering from tuberculosis for some time, and been fully conscious that his time was short. In connection with a recent communication for the Journal, he says: "Change the headings as you please, for my life is but for a few days." He preferred to continue in the service until released by death, and has been a faithful and devoted physician, discharging the duties of his office with ability and fidelity up to the last days of his life, until failing strength and approaching death prevented longer service. He was for some time in charge of Union Chapel Hospital, in which position he gained great praise for the ability with which he discharged his duties. We hear of the sad event just as we are going to press, and as there is to be a meeting of the physicians of the city to take action upon the occasion, we shall delay further remarks until our next issue, when suitable notice will be taken of his death.

